# Draft genome sequences of three *Lactococcus lactis* strains with multiple bacteriocin gene clusters isolated from sourdough samples

**DOI:** 10.1128/mra.01256-25

**Published:** 2025-12-29

**Authors:** Belay Tilahun Tadesse, Jennifer C. Molloy, Shuangqing Zhao, Emmelie Joe Freudenberg Rasmussen, Peter Ruhdal Jensen, Christian Solem

**Affiliations:** 1 National Food Institute, Technical University of Denmark 5205, Lyngby, Denmark; 2 Department of Chemical Engineering and Biotechnology, University of Cambridge 2152, Cambridge, United Kingdom; Nanchang University, Nanchang, Jiangxi, China

**Keywords:** *Lactococcus lactis*, bacteriocin gene, sourdough, genome sequence

## Abstract

We report on the draft genome sequences of *Lactococcus lactis* strains A4_4, C1_19, and C8_26 isolated from sourdough. The strains were found to harbor multiple bacteriocin gene clusters, where one encoding nisin Z was found in all three strains.

## ANNOUNCEMENT

Nisin, a bacteriocin produced by *Lactococcus lactis*, is commonly used as a bio-preservative in foods (1). Three *Lactococcus lactis* strains, A4_4, C1_19, and C8_26, were isolated from sourdough and demonstrated to possess a broad-spectrum antibacterial activity. The three strains were isolated from sourdough samples collected at the Technical University of Denmark, 55°47′25.79″ N and 12°31′32.06″ E. The strains were isolated and cultivated in M17 broth supplemented with 1% glucose (GM17). Genomic DNA was extracted from early stationary-phase cultures grown in 50 mL GM17 broth under static conditions (30°C, 24 h). DNA isolation was performed using the DNeasy Blood and Tissue Kit (Qiagen, Germany) from 1 mL of culture. The quality and quantity of the extracted DNA were evaluated using the Agilent 5400 system (Agilent Technologies, CA, USA).

Genomic DNA was fragmented and subjected to end-repair, A-tailing, and ligation of Illumina adapters using the Illumina DNA Prep Library Preparation Kit (Illumina, CA, USA). Adapter-ligated fragments were size-selected, PCR-amplified, and purified using AMPure XP beads (Beckman Coulter, MA, USA). Library quality was assessed using a Fragment Analyzer (Agilent, CA, USA), and quantification was performed using Qubit (Thermo Fisher Scientific, MA, USA) and qPCR. Sequencing was conducted on the Illumina NovaSeq X Plus platform (paired-end 150 bp) at Novogene (Munich, Germany). The sequencing was performed with a read length of 150 bp. Details regarding the total number of reads and sequencing depth are provided in Table 1. Quality control of the raw reads was conducted using fastp v1.0 (2). Genome assembly was carried out using Unicycler v0.5.0 (3). To evaluate the completeness and contamination of the genome bins, CheckM v1.2.4 (4) was employed. The assembled genomes were annotated using Prokka (v1.14.6) (5). The three strains exhibited 100% identity to *L. lactis*, as revealed by a PubMLST (6) analysis. The digital DNA-DNA hybridization (dDDH) percentages calculated via the Type Strain Genome Server (TYGS) (7) of strains A4_4, C1_19, and C8_26 compared to reference strains *L. lactis* ATCC 19435 showed 69.3, 76.5, and 76.3% respectively. Genome mining of *L. lactis* strains using antiSMASH v8.0 (8) and BAGEL4 (9) identified multiple bacteriocin gene clusters, where one encoding nisin Z was found in all strains and nisin Z was detected in culture media by mass spectrophotometry at the University of Cambridge. Besides the nisin Z biosynthesis cluster, strain A4_4 carried a biosynthesis cluster for enterolysin A; C1_19 carried one for lactococcin B; and C8_26 carried two additional clusters for lactococcin A and enterolysin A. The genome did not harbor any detectable virulence and antimicrobial resistance genes as screened using ABricate (Galaxy v.1.0.1) (10). *L. lactis* strain C8_26 possessed two plasmids, pSMA198 and pGdh442. All software tools were used with default parameters.

**TABLE 1:** Genome assembly and annotation statistics for *L. lactis* strains

Strains	Total reads	Coverage (×)	Number of contigs	Genome size (bp)	N50	GC content	CDS	rRNA genes	tRNA
A4_4	7,257,222	447.09	18	2,434,799	422,172	34.9	2,319	4	49
C1_19	6,955,150	395.58	32	2,637,265	328,004	34.73	2,559	4	52
C8_26	6,324,422	380.29	48	2,494,535	148,252	34.9	2,468	4	54

## Data Availability

The genome sequence of the *L. lactis* strains has been archived in NCBI under BioProject accession number PRJNA1348895. The raw Illumina sequencing data are available in the Sequence Read Archive (SRA) of strains A4_4, C1_19, and C8_26 under accession numbers SRR35876909, SRR35876908, and SRR35876907, respectively. Additionally, the assembled genomes have been submitted to GenBank with accession numbers JBSSQB000000000, JBSSQA000000000, and JBSSPZ000000000, respectively.
